# Vitamina D: síntesis, deficiencia, polimorfismos y resistencia a su acción en los países latinoamericanos

**DOI:** 10.7705/biomedica.7477

**Published:** 2024-03-31

**Authors:** Pedro Nel Rueda-Plata

**Affiliations:** 1 Médico internista y endocrinólogo Colombia

La vitamina D es un secoesteroide y una hormona cuya fuente principal (90 %) es la producción endógena en la piel, bajo la estimulación de la radiación ultravioleta B (290 a 330 nm) por una vía no enzimática. Además, las vitaminas D_2_ (ergocalciferol) y D_3_ se pueden obtener en la alimentación (10 %) a partir de algunos pescados grasos, fuentes vegetales o alimentos enriquecidos (lácteos, jugos), aunque en baja proporción. La vitamina D_3_ es hidroxilada por el hígado a 25-hidroxivitamina D, o calcidiol, que es la forma de depósito más importante. La 25-hidroxivitamina D es también la principal forma circulante y está unida a la proteína de unión a la vitamina D. El complejo 25-hidroxivitamina D con la proteína de unión refleja el estado de suficiencia de vitamina D durante las últimas cuatro semanas.

El calcidiol sufre una segunda hidroxilación en el riñón por la enzima 1-alfa hidroxilasa para convertirse en 1,25-dihidroxivitamina D, o calcitriol, que es la forma más activa que se une al receptor celular para realizar la acción hormonal. El calcitriol también se produce en otros tejidos mediante regulación paracrina ^1^.

Tanto la síntesis de calcitriol como la del metabolito inactivo 24,25-dihidroxivitamina D en el túbulo renal proximal, están reguladas por:

1. la hormona paratiroidea (PTH), que estimula la 1-alfa hidroxilación,
2. la 1,25-dihidroxivitamina D de forma paracrina, y
3. el factor de crecimiento de fibroblastos óseos (*fibroblast growth factor*-23, FGF23) producido por los osteocitos, lo que disminuye la hidroxilación 1-alfa y mejora la hidroxilación 24-alfa. La 1,25-dihidroxivitamina D también inhibe la secreción de PTH por el mecanismo de retroalimentación. El calcio y el fósforo séricos son los principales moduladores de la actividad 1-alfa hidroxilasa y 24,25-hidroxilasa, al actuar sobre la PTH y el FGF23. La hipocalcemia y la hiperfosfatemia aumentan la secreción de PTH y, en forma indirecta, estimulan la hidroxilación alfa. Simultáneamente, la 1,25-dihidroxivitamina D promueve la producción de FGF23 y, de esta manera, también la excreción urinaria de fosfato.

Todo el sistema de regulación tiende a mantener la concentración sérica de calcitriol dentro de los valores fisiológicos normales. El calcitriol sérico solo disminuirá cuando haya una deficiencia grave de vitamina D.

Es importante destacar el papel que tiene el magnesio en los diferentes pasos de la síntesis de vitamina D, pues las enzimas de hidroxilación renal y hepática para producir 25-dihidroxivitamina D y 1,25-dihidroxivitamina vitamina D dependen del magnesio como cofactor; la unión a su proteína de transporte y la actividad de la vitamina D también dependen de este elemento. Por tanto, en presencia de déficit de magnesio, la síntesis, el transporte y la activación de la vitamina D se reducen. El magnesio también juega un papel crítico en la síntesis y liberación de la PTH, y la inhibición de esta por la vitamina D. Los efectos combinados de la deficiencia de magnesio y de vitamina D pueden conducir a resultados clínicamente relevantes, como un mayor riesgo de fracturas por fragilidad, particularmente, en mujeres ^1^^,^^2^.

Otro aspecto importante, y que a su vez es motivo de controversia, es la determinación de las concentraciones óptimas de esta hormona en sangre. La suficiencia de vitamina D se evalúa midiendo la concentración sérica total de 25-dihidroxivitamina D, que corresponde a la suma de los niveles séricos de 25-hidroxivitamina D_3_ y 25-hidroxivitamina D_2_. La espectrometría de masas en tándem se considera el método más preciso para medir la 25-dihidroxivitamina D y sus metabolitos. A pesar de esto, los inmunoensayos automatizados que miden la 25-dihidroxivitamina D total en suero todavía se utilizan de forma rutinaria en muchos laboratorios clínicos. El calcitriol, o 1a,25-dihidroxicolecalciferol, solo se mide si hay una conversión defectuosa de 25-dihidroxivitamina D en 1a,25-dihidroxicolecalciferol debido a insuficiencia renal, hipoparatiroidismo, algunas formas genéticas de raquitismo y osteomalacia oncogénica ^3^^,^^4^.

En la mayoría de las guías se considera que los niveles óptimos de 25-dihidroxivitamina D son iguales o mayores de 30 ng/ml, con base en argumentos como los siguientes: a partir de este nivel, se logra la máxima disminución de las concentraciones séricas de PTH, aún en personas mayores de 65 años; la absorción intestinal de calcio se incrementa en el 40 al 65 % en mujeres menopáusicas al pasar la 25-dihidroxivitamina D de 20 a 32 ng/ml, y se relaciona con la reducción de eventos clave como la incidencia de fracturas de cadera desde los 18 meses en mujeres de 60 a 106 años, cuando se suplementa con vitamina D_3_; además, reduce la PTH sérica y se alcanza una concentración sérica media de 25-dihidroxivitamina D de 41 ng/ ml, y este efecto se sostiene, al menos, por tres años. Se recomienda para la salud ósea que estos valores se mantengan en el rango entre 30 y 40 ng/ml, que son las concentraciones circulantes que logran la máxima supresión de PTH y evitan el hiperparatiroidismo secundario ^4^^,^^5^.

La deficiencia de vitamina D se ha relacionado con disminución de la absorción de calcio en la dieta, lo que lleva a mayor expresión de la hormona paratiroidea, posterior resorción ósea y disminución de la masa esquelética ^6^. Aún existen controversias sobre la suplementación de vitamina D para mantener la salud ósea, particularmente en lo que respecta a los niveles óptimos de 25-hidroxivitamina D para iniciar el tratamiento ^7^. Es más, la ausencia de recomendaciones específicas para Latinoamérica resalta la necesidad de tener en cuenta aspectos únicos de los sistemas de salud de la región y la diversidad de sus comunidades ^8^^,^^9^.

La exposición segura al sol y la ingestión en la dieta han sido tradicionalmente las principales recomendaciones para la población general ^10^. Sin embargo, en Latinoamérica, se presentan desafíos para alcanzar los niveles deseados de estas fuentes naturales. Se llevó a cabo una revisión sistemática con el objetivo de arrojar luz sobre el grado de deficiencia de vitamina D en Latinoamérica y el Caribe, y se encontró que la deficiencia de vitamina D en algunos países podría considerarse inexistente como un problema de salud pública (prevalencia < 5 %) o categorizarse como un problema leve de salud pública (prevalencia entre el 5 y el 19,9 %). Sin embargo, los datos revelaron que, en la mayoría de los países, la deficiencia de vitamina D se clasifica como un problema de salud pública de leve a moderado (prevalencia entre el 20 y el 39,9 %) y, en algunos casos, incluso como problema grave de salud pública (prevalencia igual o mayor del 40 %).

En cuanto a la ingestión en la dieta, un estudio realizado en niños y adolescentes brasileños que mostraban signos de retraso en el crecimiento reveló que solo el 36 % de estos jóvenes tenía una ingestión adecuada de vitamina D. De manera similar, un estudio en Costa Rica informó que la ingestión media de vitamina D en adolescentes y adultos jóvenes de 12 a 20 años, era de 185 ± 7,6 UI, por debajo de lo recomendado por la Academia Nacional de Medicina de Costa Rica (ACANAMED) ^11^^,^^12^.

Se recomienda la exposición segura al sol como método natural complementario para lograr niveles adecuados de vitamina D. Nuestros países tienen grandes variaciones en las condiciones geográficas, las cuales también influyen en la síntesis de vitamina D. En el estudio D-SOL [interacción entre la suplementación con vitamina D y la exposición a la luz solar en mujeres (*Living in Opposite Latitudes*)], se investigaron los efectos del suplemento de vitamina D y la exposición a la luz solar sobre la concentración de vitamina D en mujeres brasileñas que viven en diferentes latitudes Un análisis transversal de los datos de referencia de este estudio doble ciego, aleatorizado y controlado con placebo demostró la influencia significativa de la ubicación geográfica sobre el estado de la vitamina D. Más específicamente, se encontró que las mujeres brasileñas residentes en Inglaterra (latitud 51° N), tenían una concentración sérica media notablemente más baja de 25-dihidroxivitamina D, en comparación con las que viven en Brasil (latitud, 16°S).

Para lograr una concentración de 25-dihidroxivitamina D de 30 ng/ml, se determinó que la exposición a la radiación UVB necesaria era de 2,2 SED (1 SED = 100 J/m^2^). El análisis de los datos completos del estudio D-SOL también reveló que una dosis diaria de 600 UI era eficaz para aumentar y mantener niveles adecuados de vitamina D, independientemente de la latitud y la exposición a la luz solar, correlacionados positivamente con la concentración inicial de 25-dihidroxivitamina D ^13^^,^^14^. Estos hallazgos subrayan la importancia de la ubicación geográfica y de la exposición solar asociada, y sugieren que la suplementación con vitamina D podría mejorar eficazmente la concentración sérica de la vitamina D, independientemente de la ubicación geográfica y de la exposición a los rayos UVB.

En el presente número de *Biomédica*, Caroline de Souza Silverio y Carolina Bonilla ^15^ hacen un excelente aporte al conocimiento de las relaciones de los genes con los niveles de la vitamina D en la población de Brasil. Encontraron que el gen *GC* mostró la asociación más fuerte con los niveles de vitamina D, lo que coincide con los hallazgos existentes en los europeos. Además, destacan que el gen *VDR* era el más investigado, a pesar de no estar asociado con la vitamina D en *genome-wide association studies* (GWAS). Concluyen que se necesitan más investigaciones para validar los indicadores de los niveles de vitamina D en la población de Brasil, dando prioridad, por ejemplo, al *GC* en lugar de al *VDR*.

En este sentido, en una revisión sistemática desarrollada por Simões *et al*. sobre los polimorfismos del receptor de vitamina D y sus variantes (BsmI, ApaI, TaqI y FokI), se encontró que pueden modificar la concentración sérica de vitamina D y, de esta manera, influir en el desarrollo y la gravedad de diferentes enfermedades ^16^. En Brasil, estos autores evaluaron la presencia de polimorfismos en osteoporosis, degeneración del disco intervertebral, enfermedades infecciosas, enfermedades autoinmunitarias y síndrome de ovario poliquístico, y obtuvieron resultados controvertidos, que atribuyen al profundo mestizaje y la diversidad étnica de la población brasileña. Sin embargo, algunos estudios señalan, más claramente, la importancia de estas variantes. Por tanto, es de suma importancia que se realicen más estudios con grupos más grandes de pacientes, con el fin de dilucidar el papel de las variantes de *VDR* en las diferentes enfermedades, lo que puede ayudar, en el futuro, al pronóstico y tratamiento de los pacientes. Tomando en conjunto estos resultados con los hallazgos de Silverio y Bonilla, las recomendaciones sobre darle más importancia a los genes, especialmente el *GC*, cobran mayor relevancia.

Finalmente, otros factores que pueden afectar la reacción a la vitamina D son la resistencia adquirida a la vitamina D, como fue hipotetizado en 2013 por el profesor Cicero Coimbra en São Paulo (Brasil). Esta se presenta de una forma no hereditaria con características distintivas de la resistencia adquirida a la vitamina D, en la que se observa una concentración elevada de PTH pero niveles de 25-dihidroxivitamina D en el rango ideal. Y, por lo tanto, indican una producción suficiente de 25-dihidroxivitamina D_3_, pero con niveles elevados de PTH no correspondientes con los esperados para los de vitamina D.

Para mayor claridad, me permito explicarlo de esta manera: el nivel óptimo recomendado de 25-dihidroxivitamina D_3_es mayor de 40 ng/ml y debería correlacionarse fisiológicamente con una concentración de PTH en la mitad del tercio inferior de su valor específico del rango de referencia de laboratorio; si éste es de 15 a 65 ng/ml, el tercio inferior del rango sería de 15 a 31 y la mitad de su tercio inferior sería de 23 ng/ml. De tal manera que para este valor de vitamina D, los niveles de PTH por encima de 23 ng/ml estarían relativamente elevados y harían sospechar la resistencia a la vitamina D ^17^.

En la etiología de la resistencia adquirida a la vitamina D, se encuentra que los polimorfismos de los genes dentro del sistema de la vitamina D constituyen la base de una sensibilidad a desarrollar dicha resistencia y, por tanto, a enfermedades autoinmunitarias. Los bloqueos parciales del receptor de vitamina D por agentes patógenos, glucocorticoides (estrés crónico) y, supuestamente, toxinas ambientales como los metales pesados, pueden interactuar con tal sensibilidad, de modo que surja una resistencia a la vitamina D.

Por último, la baja exposición al sol y el envejecimiento -que también se correlacionan con enfermedades autoinmunitarias- exacerban aún más esta situación. La importancia de esta hipótesis es que se asocia con la presencia de enfermedades autoinmunitarias y la aplicación del protocolo Coimbra ^18^ por personas suficientemente entrenadas para administrar dosis elevadas de vitamina D que conduce a la mejoría de estas enfermedades. Este concepto que, aunque puede ser controvertido por algunos, permite hacer una evaluación de diferentes situaciones clínicas y, también, abre la posibilidad de opciones terapéuticas de la vitamina D en sus acciones esqueléticas y, especialmente, en las no esqueléticas o pleiotrópicas.

La vitamina D nos permite analizar los diferentes aspectos de su comportamiento en una región tan extensa, tan variada, con diferentes altitudes, latitudes y climas, con poblaciones genéticamente heterogéneas por la variabilidad del mestizaje, y composición étnica, diferentes culturas, costumbres, y lenguas.
